# The benefits of swimming together

**DOI:** 10.7554/eLife.86807

**Published:** 2023-03-22

**Authors:** Iain D Couzin, Liang Li

**Affiliations:** 1 Department of Collective Behaviour, Max Planck Institute of Animal Behavior Konstanz Germany; 2 Centre for the Advanced Study of Collective Behaviour, University of Konstanz Konstanz Germany; 3 Department of Biology, University of Konstanz Konstanz Germany

**Keywords:** fish schooling, robotics, in-line swimming, thrust wakes, fluid dynamics, Other

## Abstract

When a fish beats its tail, it produces vortices in the water that other fish could take advantage of to save energy while swimming.

**Related research article** Thandiackal R, Lauder GV. 2023. In-line swimming dynamics revealed by fish interacting with a robotic mechanism. *eLife* **12**:e81392. doi: 10.7554/eLife.81392.

Around half of all fish species spend a portion of their lives as part of a group or ‘school’ (Shaw, 1978). Swimming together offers a wide range of benefits, such as improved sensing, decision-making and navigation (Berdahl et al., 2013). It has also long been thought that moving in a school could reduce the energy expended by the fish as they swim. However, proving that swimming with others does save energy – and, if it does, how – has been challenging.

In many species, the tail of the fish produces a pattern of swirling vortices when it beats, with each vortex rotating in the opposite direction to the one that came before. The water in these ‘thrust wakes’ exhibits, on average, faster flow than the water in free streams in which no fish or other objects are present. A similar phenomenon occurs when water flows past a physical object; in this case, however, the pattern of vortices is different and results in ‘drag wakes’, and the average speed of water (with respect to the flow direction) is slower than it is in a free stream (Figure 1).

**Figure 1.**
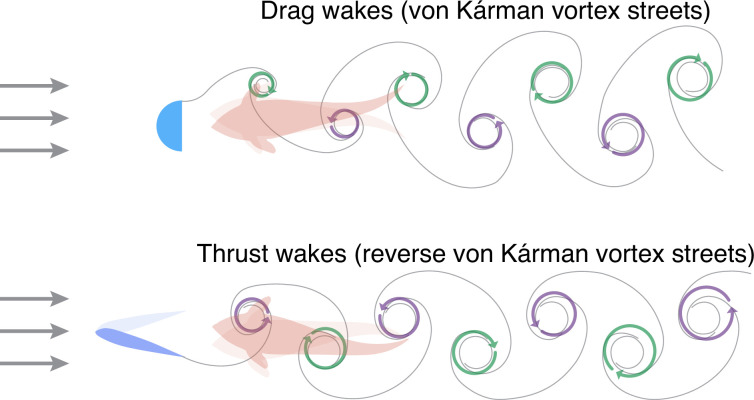
Swimming in drag wakes and thrust wakes. When water flows (grey arrows) past a physical object (light blue; top), it forms a pattern of swirling vortices in which the water rotates in either a clockwise direction (green arrows) or an anticlockwise direction (purple arrows). The arrangement of the vortices (which is called a ‘drag wake’ or a von Kárman vortex street) means that a fish swimming towards the object encounters water that is flowing slower on average than it would be if the object was not there. When a fish beats its tail (blue; bottom), it also produces a pattern of swirling vortices. In this case, however, the arrangement of the vortices (which is called a ‘thrust wake’ or a reverse von Kárman vortex street) means that a second fish swimming behind the first fish encounters water that is flowing faster on average than it would be if the first fish was not there. Thandiackal and Lauder used a hybrid robotic-animal experimental set-up to study fish swimming in thrust wakes.

Image credit: Iain D Couzin and Liang Li (CC BY 4.0).

Previous studies have shown that fish can save energy by synchronising the wave-like motion of their body, also known as undulations, to drag wakes (Liao et al., 2003). Remarkably, even dead fish can synchronise in this way and use the reduced flow of oncoming water to continue moving upstream, albeit momentarily (Beal et al., 2006). It has been proposed that thrust wakes also benefit fish in a similar way, but direct experimental evidence in support of this idea has been lacking (Maertens et al., 2017; Kurt and Moored, 2018). Now, in eLife, Robin Thandiackal and George Lauder from Harvard University report the results of experiments on fish swimming in thrust wakes (Thandiackal and Lauder, 2023).

Since it is not practical to produce well-controlled vortices using real fish, the researchers turned to an elegant engineering solution to study what happens when one fish swims behind another: they replaced the fish in front with a robotic flapping foil that can produce vortex patterns comparable to those produced by a real fish (see also Harvey et al., 2022), and then introduced a live brook trout (*Salvelinus fontinalis*) into the flow tank. Thandiackal and Lauder observed that this fish deliberately swam within the thrust wake generated by the foil. This suggests that unlike cyclists – who cycle behind one another to benefit from the reduced air flow in the drag wake – fish deliberately move into regions where the flow of water is higher rather than lower.

When swimming in a thrust wake the trailing fish synchronises its body undulations to the pattern of vortices. The specific timing of the undulations, together with a detailed analysis of the fluid flow patterns around the fish, suggests they are exhibiting a strategy termed vortex phase matching – an energy-saving behaviour that has also been observed in pairs of real fish (Li et al., 2020). Fish rely on a system called the lateral line organ to detect changes in the water around them but, surprisingly, the study of pairs of real fish found that they did not rely on this organ to perform vortex phase matching: rather, they appeared to rely on proprioception (that is, signals from neurons within their muscles and joints).

This study by Thandiackal and Lauder goes further than the work of previous groups in multiple ways. First, it shows that the oscillating motion of the head of the trailing fish allows it to intercept oncoming vortices in way that reduces the average force exerted by the water that has been pushed over its head (termed pressure drag). The researchers suggest that this is achieved through the motion of the fish’s head allowing them to ‘catch’ the vortices better, which is consistent with previous results (Maertens et al., 2017; Harvey et al., 2022). Second, their work provides the first quantitative visualisation of the hydrodynamics around fish during vortex phase matching. The data suggest that a fish exploits thrust wakes by allowing the energy from the vortices to ‘roll’ down both sides of its body when it is directly behind the foil, or only roll down one side when the fish is slightly to one side rather than directly behind. Together these effects mean a fish swimming behind the foil can maintain a certain speed while beating its tail less frequently than it would have to do if it were not swimming behind the foil. This suggests the fish needs to use less energy than if it were swimming alone.

By developing a precise mechanistic framework, Thandiackal and Lauder provide new insights into why some fish swim one behind another. Furthermore, their hybrid robotic-animal approach offers exciting opportunities for future research; for example, it could help researchers to evaluate other swimming configurations, and to study what happens when fish transition from swimming in pairs to swimming in larger schools. It could also be used to measure the energy expenditure associated with different swimming strategies more directly by incorporating physiological measurements, such as heart rate. It is clear that we have much more to learn about why fish tend to swim together.
